# The effect of transcranial direct current stimulation and inhibitory control training on depression and anxiety among post-stroke individuals

**DOI:** 10.1186/s12883-025-04042-6

**Published:** 2025-01-27

**Authors:** Csaba Kazinczi, Noemi Szepfalusi, Viola Luca Nemeth, Adrienn Holczer, Katalin Jakab, Laszlo Vecsei, Peter Klivenyi, Anita Must, Mihaly Racsmany

**Affiliations:** 1 Department of Neurology, University of Szeged, 6, Semmelweis Street, Szeged, 6725 Hungary; 2 Department of Clinical Psychology, Semmelweis University, 25, Üllői Street, Budapest, 1091 Hungary; 3 Department of Education and Psychology, Faculty of Social Sciences, University of Atlántico Medio, Las Palmas de Gran Canaria, Spain; 4 HUN-REN-SZTE Neuroscience Research Group, Hungarian Research Network, University of Szeged (HUN-REN-SZTE), Danube Neuroscience Research Laboratory, Tisza Lajos krt. 113, Szeged, 6725 Hungary; 5 HUN-REN-SZTE Neuroscience Research Group, University of Szeged, Neuroscience Research Group; 6, Semmelweis Street, Szeged, 6725 Hungary; 6 Department of Psychiatry, Whanganui District Health Board, 100 Heads Road, Whanganui, 4501 New Zealand; 7 University of Szeged, Institute of Psychology, 2, Egyetem Street, Szeged, 6722 Hungary; 8 Institute of Cognitive Neuroscience and Psychology, HUN-REN Research Centre for Natural Sciences, 2, Magyar Tudósok Boulevard, Budapest, 1117 Hungary; 9 Cognitive Medicine Research Group, Competence Centre for Neurocybernetics of the Life Sciences Cluster, Centre of Excellence for Interdisciplinary Research, Development and Innovation, University of Szeged, 13, Dugonics Square, Szeged, 6720 Hungary

**Keywords:** TDCS, Computer-based cognitive training, Depression, Anxiety

## Abstract

**Background:**

Recent research has highlighted the role of fronto-parietal brain networks and cognitive control in mood disorders. Transcranial direct current stimulation (tDCS) and computer-based cognitive training are used in post-stroke rehabilitation. This study examined the combined effects ofof computer-based inhibitory control training (ICCT) and anodal tDCS on post-stroke depression and anxiety.

**Methods:**

Thirty-five participants were randomly assigned to one of three groups: active tDCS treatment (A), sham tDCS treatment with ICCT (T), or active tDCS with ICCT (AT), for a duration of ten days. Primary outcome measures included the Beck Depression Inventory (BDI), Hamilton Depression Rating Scale (HAM-D), and Spielberger’s State-Trait Anxiety Inventory (STAI-S/T). Statistical analysis was performed using a Mixed-model Analysis of Variance, with supplementary Bayesian analysis.

**Results:**

The AT group showed a significant improvement in BDI scores (*p* < .001), whereas no significant effects were observed on the HAM-D, STAI-T, or STAI-S scales.

**Conclusions:**

The combination of tDCS and ICCT reduced depressive symptoms as measured by the BDI; while no significant effects were found with either treatment alone. Further research is needed to explore the mechanisms behind the synergistic effects in the treatment of post-stroke mood disorders.

**Supplementary Information:**

The online version contains supplementary material available at 10.1186/s12883-025-04042-6.

## Introduction

 Stroke can lead to cognitive impairments (e.g., memory and attention) and is also associated with changes in affective factors, commonly resulting in post-stroke depression (PSD), which has a prevalence of 31.1%, and post-stroke anxiety (PSA), with a prevalence of 20.4% [1–4]. PSD and PSA negatively impact the quality of life and social interactions [5, 6]; however, neurorehabilitative interventions can improve post-stroke cognitive and mood factors effectively [7]. Current rehabilitation methods require substantial resources, highlighting the need to explore alternative approaches. Transcranial direct current stimulation (tDCS) and computer-based cognitive training (CCT) programs have low resource intensity and are widespread tools in post-stroke rehabilitation. While tDCS seems to be effective in treating mood-related symptoms after stroke [8]; research on CCTs is relatively limited, possibly due to differing goals and mechanisms of action [9, 10].

Regarding the primary mechanism of action, tDCS is a non-invasive neuromodulation technique that uses direct current to modulate neuronal excitability by altering membrane potential, with anodal stimulation facilitating activity and cathodal stimulation inhibiting it [11, 12]. Hence, tDCS is believed to modulate neuronal excitability and induce synaptic plasticity, effects that can be achieved through a series of therapeutic treatments. These outcomes largely depend on factors, such as current density, electrode placement, stimulation duration, session frequency, and target location [13]. Depending on the target location and stimulation parameters, recent studies suggest an impact of tDCS on neural circuits involved in mood regulation [14]. Furthermore, stimulating the dorsolateral prefrontal cortex (DLPFC) has been shown to reduce negative emotions, increase cortical excitability, and enhance synaptic plasticity, supporting the use of tDCS [12, 15]. In addition to the DLPFC, the fronto-parietal network (FPN) plays a crucial role in mood regulation. This was highlighted in a meta-analysis by Kaiser and colleagues (2015), which found that depression is associated with decreased connectivity within this network [12, 16]. Similarly, Sylvester and colleagues (2012) identified dysfunction in the fronto-parietal network (FPN) in cases of anxiety, suggesting that these regions may be appropriate targets for tDCS stimulation. Neurological conditions such as stroke can profoundly affect this network [17]; with PSD and PSA being integrally associated with these changes [18]. Furthermore, promising results have been observed in the treatment of PSD [18]; for example, Valiengo and colleagues (2017) reported significant improvements on the Hamilton Depression Rating Scale after tDCS treatment [4].

A transdiagnostic approach emphasizes the shared connections between depression and anxiety, suggesting that targeting cognitive control (CC) mechanisms could be an effective treatment strategy [19]. Research by Sylvester et al. (2012) and Kaiser et al. (2015) also indicates that fronto-parietal structural network dysfunction impairs CC, which is crucial for selecting thoughts and emotions in response to tasks and social context [20]. Advances in brain connectivity research have identified six key networks involved in cognitive control (CC), with the FPN highlighted as one of the most significant [20]. This approach may be particularly relevant for treating psychiatric disorders such as depression, where stimulation of the DLPFC within the FPN is utilised to modulate distal brain regions that influence mood [21]. In addition, Egorova and colleagues (2017) found that PSD is linked to reduced connectivity between the left DLPFC and right supramarginal gyrus, suggesting depressive symptoms post-stroke stem from network impairments rather than direct lesions. Impaired cognitive control (CC) significantly correlates with depressive symptoms, highlighting CC’s critical role in mood regulation and its impact on managing negative emotions, particularly post-stroke [22, 23].

During rehabilitation, cognitive control training (CCT) offers a potential method to enhance functions associated with cognitive control [24]. The core mechanism of CCT lies in its ability to produce therapeutic effects on functions and tasks directly associated with the trained domain (near transfer) as well as on those not directly linked to it (far transfer) [25]. Cognitive control (CC) training through CCT encompasses skills such as attention, working memory, and inhibition [26]; which are vital for emotion regulation and mitigating depressive and anxiety symptoms [27]. Research on post-stroke depression by Jaywant and colleagues (2022) underscores the importance of executive functions in managing attentional resources and suppressing irrelevant information [28]. Furthermore, inhibition is crucial for regulating depression and other mood-related factors [29–31].

The assessment and modulation of inhibitory and cognitive control (CC) functions can be conducted using various tests, with “Flanker tasks” being among the most prominent over the past few decades [32]. In this task, participants are presented with a central target stimulus, flanked by distracting stimuli. The goal is to focus on the target while inhibiting responses to the surrounding, potentially conflicting information [32]. However, as the study by Geest and Engelbregt (2022) points out, it is a procedure explicitly used in testing, and its use as cognitive training is not widespread. However, as highlighted by Geest and Engelbregt (2022), this procedure is primarily used for testing purposes and has not been widely adopted as a method for cognitive training. Nonetheless, the Flanker task could potentially serve as a cognitive training tool for developing cognitive control (CC) alongside its diagnostic applications. For example, a recent study by Grützmann and colleagues (2022) demonstrated that using the Flanker task in a structured training program may improve interference control and certain aspects of emotional processing, suggesting its potential in mood regulation therapies. One advantage of using the Flanker task, alongside other cognitive training tasks (e.g.,the “n-back task”), is that it provides an opportunity to explore various methods for assessing the transfer effects of inhibition training to improve mood regulation, particularly in post-stroke patients. Additionally, combining tDCS with CCT has the potential to enhance mood regulation and reduce symptoms. This approach could lead to more effective and lasting therapeutic outcomes compared to conventional treatments alone [33, 34].

This study aimed to enhance mood regulation by training inhibitory functions using ICCT (a modified Flanker task), which directly engages cognitive control processes, and by applying anodal tDCS to areas within the FPN, including the DLPFC, following the work of Sylvester et al. (2012) and Kaiser et al. (2015). This intervention combines both neural and cognitive stimulation potentially leveraging synergistic effects by targeting both neural and behavioural mechanisms that support mood and anxiety regulation. In this regard, our investigation is novel as tDCS, or this specific type of cognitive training program has not been studied in post-stroke patients within this design. We hypothesised that modulation of inhibition, as a key aspect of cognitive control combined with anodal stimulation of the FPN, would lead to improvement in mood and anxiety symptoms, as reflected in the scores on the questionnaires used.

## Methods

### Participants

We recruited 35 post-stroke Hungarian speaking individuals (M_age_ = 59.6; SD = 10.9) in the Neurorehabilitation Unit at the Department of Neurology, Albert Szent-Györgyi Clinical Centre. All participants received additional physiotherapy and occupational therapy on the ward. We specifically chose patients with measurable cognitive deficits on Addenbrooke’s Cognitive Examination (ACE) but retained reading and comprehension abilities. The exclusion criteria were the following: previous dementia unrelated to stroke; cerebral atrophy; comorbid major psychiatric disorders (e.g., major depression, alcohol use disorder); hemorrhagic stroke; the presence of ferromagnetic metal in the body (e.g., pacemaker or deep brain stimulator) or epilepsy. Regarding lesion location, 12 patients had right-sided lesions, 12 had left-sided lesions, and 11 had bilateral or subcortical lesions. Nineteen patients participated in our experimental therapy (see Experimental design) within three months of the stroke occurring (M = 1.29, SD = 0.87), while 16 patients have been enrolled into our experimental therapy at least three months after sufferring the lesion (M = 41.81, SD = 46.21). The study was approved by the Local Ethics Committee of the University of Szeged, and all patients provided informed consent (165/2014) and all patients provided informed consent in accordance with the Declaration of Helsinki (see Table 1 for baseline characteristics).

**Table 1:** Baseline characteristics of the sample

	Sample	AT group	A group	T group		
	Mean SD (±)	Mean SD (±)	Mean SD (±)		Mean SD (±)	Between Group Significance (*p*)
Age	59.68 11.10	54.27 12.97	63.20 9.63		61.54 9.36	0.135
Education (years)	12.03 3.40	11.36 3.10	11.60 3.13		12.92 3.88	0.493
ACE	76.31 9.89	75.45 9.88	71.50 8.37		79.92 10.00	0.084
Gender (male/female)	22/13	6/5	5/5		11/3	0.341
Stroke localization (left/right/both or subcortical)	12/12/11	5/2/4	3/5/2		4/5/5	0.626
Elapsed time after stroke (within three months/after three months)	19/16	6/5	7/3		6/8	0.435

The table summarises the demographic and clinical baseline data for the total sample and the three experimental groups (AT, A and T). Variables include age, education (years), ACE scores, sex, stroke location and time since stroke. Data are presented as mean ± SD for continuous variables and as numbers for categorical variables, with *p*-values indicating no significant difference between groups. Abbreviations: Active tDCS treatment (A), sham tDCS treatment with ICCT (T), active tDCS treatment with ICCT (AT)

### Experimental design

**Fig. 1:**
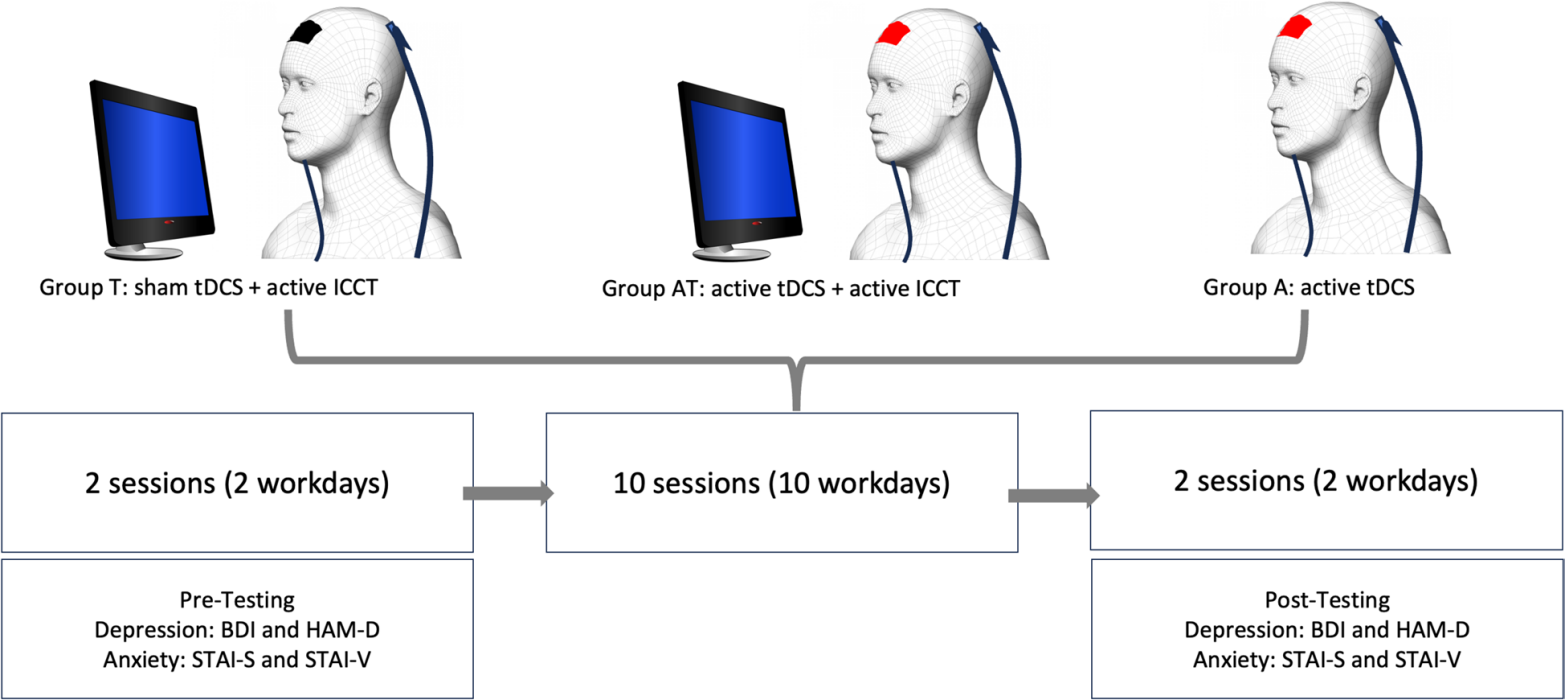
Experimental design. In the pre-testing phase (two sessions), we conducted a baseline evaluation using Beck’s Depression Inventory (BDI), Hamilton Depression Rating Scale (HAM-D), and Spielberger’s State-Trait Anxiety Scale (STAI-S and STAI-T). In the experimental phase (10 sessions), the participants were randomly allocated into three groups: A - Active tDCS only; T - Sham tDCS with ICCT; and AT - Active tDCS with ICCT. Finally, we conducted post-testing (two sessions) using the same battery of assessments as in the baseline phase

We adopted the study design based on the previous work of Martin and colleagues [35] by randomly allocating the participants into three experimental groups: Active tDCS without ICCT (A), Sham tDCS with ICCT (T), and Active tDCS with ICCT (AT). Randomisation was done using a computerised random number generator; the individuals were unaware of the specific experimental group to which they were assigned. We gathered baseline data on general characteristics (e.g., age, gender, stroke type, post-stroke duration, education) and cognitive functioning at the initiation and conclusion of the 10-session experimental program. The tDCS and ICCT training for the experimental groups consisted of 10 sessions, conducted over 10 consecutive working days. Following the 10-session intervention period, follow-up measurements were conducted on two separate days (for the experimental design, see Fig. 1). Participants were given detailed information regarding possible side effects. Participants receiving sham tDCS stimulation were unaware that they were not receiving actual stimulation; and they underwent similar preparatory steps as those in the active tDCS group (e.g., electrode positioning and placement). For the combined application in the AT group, patients received tDCS stimulation and ICCT training simultaneously, based on the work of Martin and colleagues (2014), which suggests that simultaneous (online) stimulation is more effective compared to sequential (offline) stimulation [36]. The psychological tests, participant records, cognitive training program, tDCS equipment, and research materials were securely stored in a lockable cabinet. Data collection and research administration were overseen by experienced psychologists from the Neurorehabilitation Unit of the Department of Neurology at the Albert Szent-Gyorgyi Clinical Centre.

### Primary outcome measures

#### Beck’s Depression Inventory (BDI)

The self-administered scale assesses the presence and severity of depressive symptoms. The BDI consists of 21 items, each corresponding to a specific depressive symptom. Respondents rate the severity of each symptom based on how they have felt over the past week on a scale from 0 to 3, with higher scores indicating more severe depressive symptoms. The total score is obtained by summing the scores for all items. The total score is used to categorize the severity of depression: minimal (0–13), mild (14–19), moderate (20–28), and severe (29–63). BDI has shown good reliability and validity in measuring depressive symptoms across different populations [37]. Scores above 9 indicate clinically relevant symptoms of depression.

#### Hamilton Depression Rating Scale (HAM-D)

The interviewer-administered scale is aiming to provide a standardized and systematic evaluation of the intensity and nature of depressive symptoms and consists of 17 items, each corresponding to a specific depressive symptom or behaviour, for example mood, guilt, suicidal ideation, insomnia, and weight loss. The total score is 52 points; higher scores indicate more severe depressive symptoms. The HAM-D has good reliability and validity and has been widely used in clinical and research settings. Interpreting the scores requires clinical judgment and expertise [38]. Scores above 7 indicate clinically relevant symptoms of depression.

#### Spielberger’s state-trait anxiety inventory (STAI-S/T)

This self-administered scale is designed to measure and evaluate a person’s temporary (state) and trait level of anxiety. The scale consists of 20 items scored on a 4-point Likert scale. The scores are summed to obtain a total score ranging from 20 to 80. Higher scores indicate higher levels of state/trait anxiety. The STAI-S/T has demonstrated good reliability and validity and is widely used in clinical and research settings. It has been translated into various languages, making it applicable in diverse cultural contexts. The average score for STAI-S is 38,4 (± 10,6) for men and 42,6 (± 10,8) for women; while for STAI-T is 40,9 (± 7,8) for men and 45,3 (± 7,9) for women. Values higher than one standard deviation in the positive range indicate clinically relevant symptoms [39].

In addition, we administered the following neuropsychological assessments for baseline testing: Addenbrooke’s Cognitive Examination (ACE), assessing cognitive abilities, including orientation, attention, memory, verbal fluency, language, and visuospatial abilities [40]. We used the National Adult Reading Test (NART) to assess the premorbid intellectual abilities [41].

### Additional measurements

The research study also assessed changes in cognitive function using the following measures, which will be reported elsewhere: Digit Span Forward Test, Listening Span Task, Digit Span Backward Test, Trail Making Test, and Corsi Block Tapping Task.

### Inhibitory Control Training (ICCT)

**Fig. 2:**
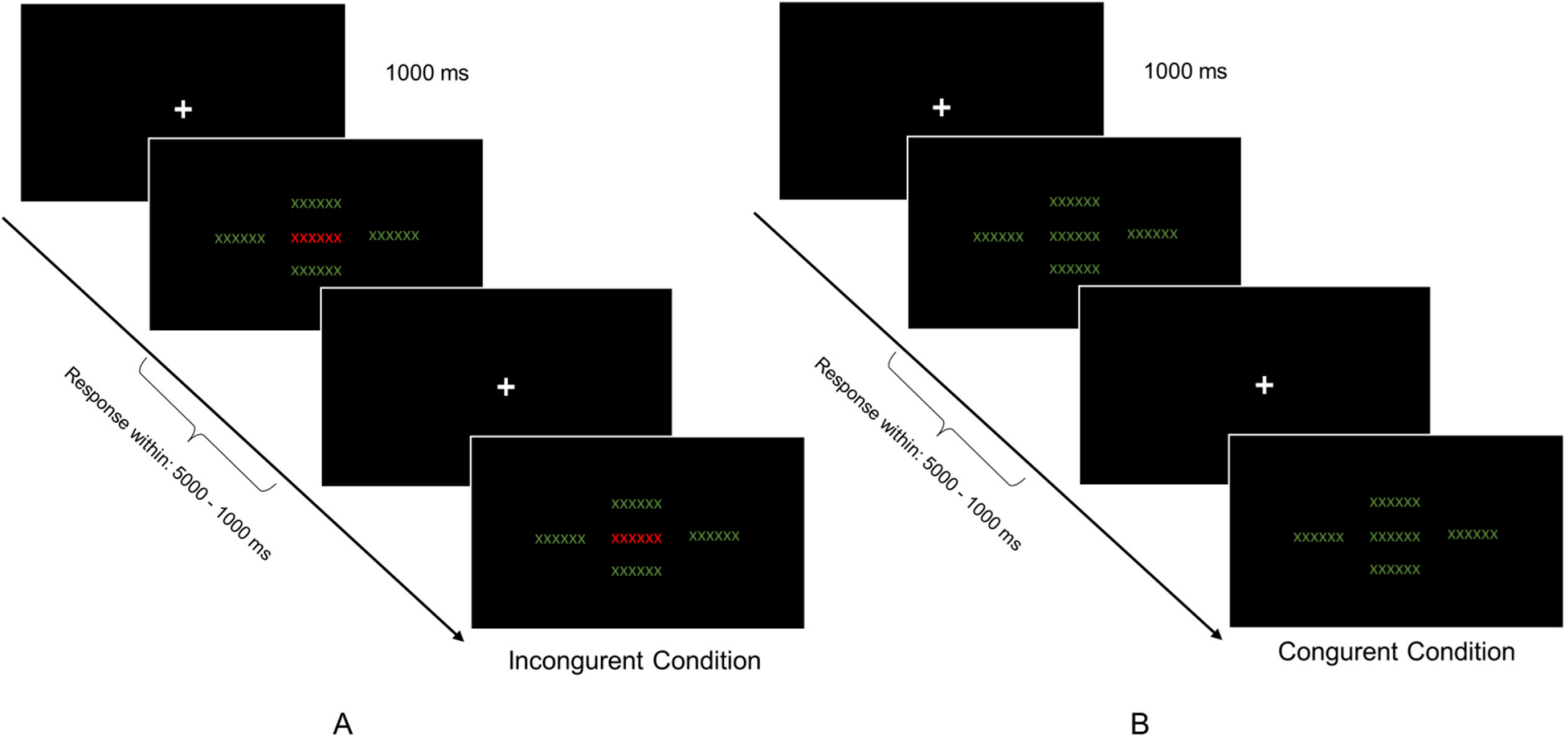
Inhibitory Control Training (ICCT). We employed Inhibitory Control Training to enhance inhibitory control functions. The training program involved two setups: incongruent (**A**) and congruent (**B**). In both cases, an identical word surrounding the middle word was displayed in red for the incongruent condition (**A**) and green for the congruent condition (**B**)

We conducted cognitive training by applying a Flanker Task to enhance inhibitory control. The task was presented with E-prime 2.0 software (Psychology Software Tools, Pittsburgh, PA). Participants were instructed to solve tasks on a screen with a dark background positioned at a distance of 75 cm. During the task, a fixation cross was presented, followed by the appearance of the target stimulus (word) at the centre of the screen in a font size of 48. Simultaneously, the same word was displayed around the target word on the left, right, below, and above, also in font size 48 and the colour green. Participants were asked to press the ‘A’ key if the surrounding words matched in color with the target, i.e.the ‘A’ key needed to be pressed if the target word was green, and the ‘L’ key had to be pressed on a Hungarian keyboard when the target word was red. In the initial session, subjects had 5000 ms to respond to each stimulus. If a participant completed the preceding block with an 80% success rate, the task’s difficulty was adjusted by reducing the reaction time to 4000, 3000, 2000, or 1000 ms, based on each subject’s previous performance. To ensure practice opportunities before each session, participants had unlimited time to familiarise themselves with the task and receive feedback. The training session encompassed 4 blocks, each containing 4 sets of 45 words. The words were matched based on frequency, length, and syllable count. The duration of the ICCT training was 720 s (12 min) (Fig. 2).

### Parameters of tDCS stimulation

**Fig. 3:**
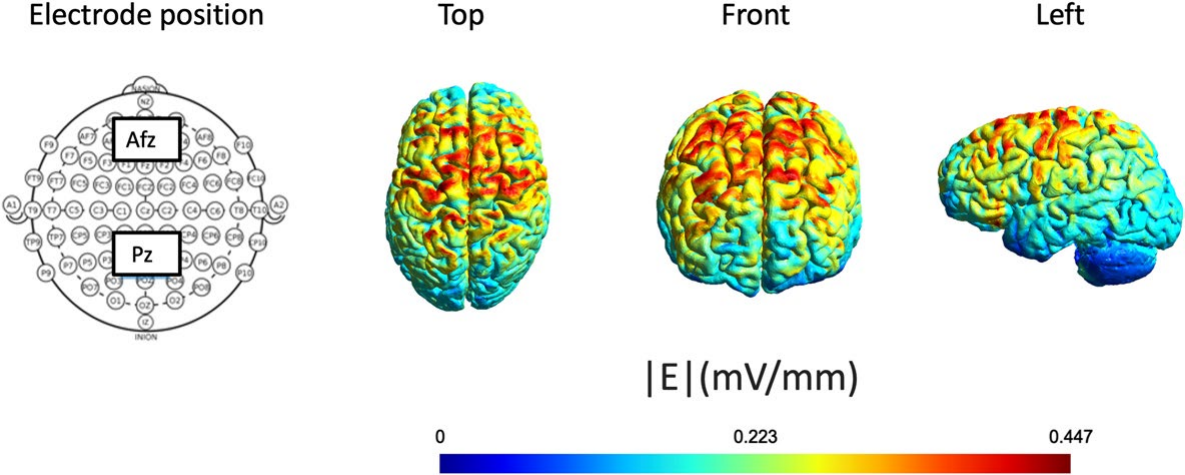
Stimulation Settings of tDCS. During active tDCS, specific cortical regions were stimulated, utilising an electrode current of 2 mA. The anodal electrode was positioned on the frontal regions (AFz), while the cathodal electrode was placed on the parietal regions (Pz)

We used the NeuroConn DC Stimulator Plus for stimulation (neuroConn GmbH, Germany). The conducted current was set at 2 mA, with a pair of surface sponge electrodes (5,5 cm x 7,5 cm). According to the international 10–20 EEG system, the anode was positioned over the ‘AFz’ and the reference electrode over the ‘Pz’ areas. Based on the previous research of Chai and colleagues (2018), we aimed to stimulate the cortex between the DLPFC and the parietal-occipital regions [42, 43]. For each participant, we first measured the head circumference and the nasion-to-inion distance using a flexible measuring tape to ensure accurate and consistent positioning. Following the internationally recognized 10–20 system, we placed electrodes along the midline of the scalp at intervals of 10%, 20%, 20%, 20%, 20%, and 10%, corresponding to the Fpz, Fz, Cz, Pz, and Oz positions, respectively [44]. The AFz position was identified as the midpoint between Fpz and Fz, while the Pz position was directly determined based on the 10–20 system. The measurements were documented for each participant, allowing precise setup replication in subsequent sessions. The nasion-to-inion distance, recorded in centimeters, was used as a reference for electrode placement during every session. To ensure consistency, we marked the electrode positions with a clinically tested, skin-friendly, and easily washable marker. This protocol minimized session variability and ensured reliable electrode placement for all participants. Furthermore, using the ‘SimNIBS 3.2 (www.simnibs.org) modelling program, we modelled the direction and strength of the current flow. The results indicated that the main stimulation fields were the frontal, parietal, and parietal-occipital cortices. In both active tDCS groups, the stimulation phase was set at 12 minutes per session, which was consistent with the duration of ICCT, similarly set at 12 minutes (720 seconds) and was defined as the optimal exercise duration for patients in the post-stroke recovery period. The AT group’s cognitive training program ran parallel to the tDCS stimulation. Experienced neuropsychologists conducted the process under the supervision of a psychiatrist and a neurologist from the Neurorehabilitation Unit of the Department of Neurology, Albert Szent-Györgyi Clinical Centre (Fig. 3).

### Statistical analysis

#### Frequentist statistical analysis

We performed the analysis using the JAMOVI Project (Version 2.3) and defined the significance at 0.05. A descriptive statistical analysis was used for demographic data, where frequency, means, and standard deviations were examined. We applied the Shapiro-Wilk normality test to determine whether the data were normally distributed. The baseline characteristics between experimental groups were examined either with frequentist One-way Analysis of Variance (ANOVA) (parametric data) or the Kruskal-Wallis Test (non-parametric data) in the case of continuous variables; contingency tables were applied for categorical data (χ^2^ test). A 2 × 2 mixed-model ANOVA was applied to examine the effect of treatment and experimental conditions. The baseline and post-test scores of psychological assessments after the ten experimental sessions were considered as [PRE-POST], while experimental conditions were defined as [CONDITION]. For statistically significant results, we examined the differences with Tukey-corrected post-hoc tests. In terms of effect size, η_p_^2^ values are interpreted as follows: less than 0.01 indicates negligible effects, 0.01–0.06 represents small effects, 0.06–0.14 corresponds to medium effects, and values above 0.14 indicate large effects. These thresholds provide a useful guide to assess the practical significance of observed effects in addition to statistical significance. Furthermore, Pearson correlation analysis was performed to examine the correlation between pre- and post-intervention scores.

#### Bayesian statistical analysis

We applied Bayesian statistics with default priors to assess the strength of the evidence in our analysis. Bayesian statistics may be an appropriate choice for dealing with possible baseline differences and sample size variances, as it allows incorporating prior information and accounting for these variances in a probabilistic framework. This approach can also provide robust results in the case of imbalances in group sizes or baseline values. A Bayesian One-way ANOVA was used to compare baseline testing, while a Bayesian Mixed-model ANOVA was used to examine the experimental design. Bayesian analyses provide estimates of evidence for either the null hypothesis (H0) or the alternative hypothesis (H1) based on the collected data. In this analysis, the null hypothesis (H0) posits that there is no effect or difference, meaning that the treatment or condition being tested has no impact on the outcome measure, and any observed differences are due to random chance or sampling error. The alternative hypothesis (H1) posits that there is an effect or a difference, meaning that the treatment or condition being tested significantly impacts the outcome measure, and the observed differences are due to the treatment or condition rather than random chance. Our analysis focused on comparing the evidence for various models against the null model. To quantify this evidence, we computed the Bayes Factor (BF10). The BF10 value indicates how much more likely the data are under one hypothesis compared to the other. The interpretation of BF10 is: BF10 < 0.1 suggests strong evidence for H0, indicating that the data strongly support the null hypothesis over the alternative hypothesis; 0.1 < BF10 < 0.33 indicates moderate evidence for H0, suggesting that the data moderately support the null hypothesis over the alternative hypothesis; 0.33 < BF10 < 1 suggests weak evidence for H0, implying that the data weakly support the null hypothesis over the alternative hypothesis; 1 < BF10 < 3 indicates weak evidence for H1, meaning the data weakly support the alternative hypothesis over the null hypothesis; 3 < BF10 < 10 indicates moderate evidence for H1, suggesting that the data moderately support the alternative hypothesis over the null hypothesis; BF10 > 10 suggests strong evidence for H1, indicating that the data strongly support the alternative hypothesis over the null hypothesis. In addition to the Bayes Factor for comparing hypotheses, Bayesian ANOVAs include the “inclusion Bayes Factor” (BFincl). The BFincl represents the relative difference between models with and without the examined effect. Essentially, BFincl measures the relative evidence in favor of including a specific effect or variable in the model compared to a model without it. The interpretation of BFincl values is similar to BF10: BFincl < 0.1 indicates strong evidence against including the effect, supporting the model without the effect; 0.1 < BFincl < 0.33 indicates moderate evidence against including the effect; 0.33 < BFincl < 1 indicates weak evidence against including the effect; BFincl = 1 indicates no preference for including or excluding the effect; 1 < BFincl < 3 indicates weak evidence for including the effect; 3 < BFincl < 10 indicates moderate evidence for including the effect; BFincl > 10 indicates strong evidence for including the effect.

#### Minimum clinically important differences

Following the recommendations outlined in the study of Masson & Tejani (2013), the calculation of the Minimal Clinically Important Difference (MCID) was conducted using a hybrid approach that integrates anchor-based and distribution-based methods. This mixed methodology ensured a robust assessment of clinically meaningful changes in participant scores. MCID calculations were performed for BDI, where frequentist and Bayesian statistical analyses indicated a significant interaction effect. Three criteria were used to classify changes as clinically important: (1) the direction of change had to indicate improvement, (2) the absolute magnitude of change had to exceed 5 points, and (3) the change had to be at least 29.64% of the baseline score [45]. These criteria combine distribution-based thresholds (absolute change and percentage of baseline) with an anchor-based assessment of the direction of improvement. In the evaluation, the degree of change was calculated as the difference between the pre-treatment and post-treatment scores for each participant. Based on these, two classifications have been made, (I) one for an improvement trend of at least 5 points with a threshold of 29.64%, and the other (II) for an improvement trend with a threshold of 29.64%. The reason for this consideration is that relative and absolute point value changes are assessed together, and relative scores are assessed separately. A χ2-square test was conducted to examine the distribution of participants meeting the MCID criteria within each group. This allowed the proportion of groups achieving clinically meaningful improvement to be assessed.

## Results

No baseline differences were observed between the experimental groups regarding the primary outcome measures (BDI, STAI-S, STAI-T), except in the case of HAM-D, where the active tDCS group showed a significantly lower score, as determined by frequentist One-way ANOVA. We found no significant differences in general cognition as measured by ACE, where patients achieved an average score of 76.3 (SD = 9.89). We tested the experimental groups for education time, age, location, and time of lesion. We found no group differences between experimental conditions. Examining distribution using the Shapiro-Wilk test showed that STAI-T and HAM-D averages were not normally distributed at the baseline. The BDI and HAM-D scores were above the normal range for the total sample, while the STAI-S and STAI-T scores were within the normal range (see Tables 1 and 2 for baseline comparisons). Furthermore, correlation analysis revealed associations between pre-and post-treatment measures of depression and anxiety. The strongest correlations were observed between post-treatment STAI-T and BDI scores, *r* = .79, *p* < .001. Pre-treatment BDI scores were also correlated with post-treatment BDI scores, *r* = .75, *p* < .001. Moderate correlations were found between pre-treatment STAI-T and post-treatment BDI scores, *r* = .43, *p* = .010. The HAM-D scale showed a correlation with both post-treatment HAM-D, *r* = .68, *p* < .001; and BDI scores *r* = .58, *p* < .001). Finally, pre- and post-treatment STAI-T scores were also significantly correlated, *r* = .65, *p* < .001 (Supplementary Table 1).

**Table 2:** Baseline and post-testing data of the sample

	Baseline (± SD)					Post-testing (± SD)			
Measure	Sample	AT group (*n* = 11)	T group (*n* = 14)	A group (*n* = 10)	Baseline Difference (*p*)	Sample	AT group (*n* = 11)	T group (*n* = 14)	A group (*n* = 10)
BDI	11.80 (8.63)	17.36 (8.95)	9.21 (6.53)	9.30 (8.69)	0.056	7.00 (7.51)	9.45 (6.88)	9.86 (8.11)	7.70 (7.90)
HAM-D	7.29 (5.92)	9.45 (6.49)	8.50 (5.84)	2.78 (2.17)	0.002*	4.86 (4.25)	6.82 (3.28)	5.00 (5.16)	2.50 (2.64)
STAI - S	40.30 (12.70)	44.50 (10.00)	39.30 (9.86)	37.20 (18.02)	0.362	36.60 (11.60)	37.90 (11.38)	35.30 (9.78)	37.10 (4.67)
STAI-T	43.00 (12.10)	46.20 (10.74)	39.40 (7.08)	36.90 (16.92)	0.075	36.80 (9.55)	37.90 (9.43)	38.10 (10.22)	33.80 (8.99)

The table presents the baseline and post-test scores for the full sample and the three experimental groups (AT, A and T) for the four outcome measures: the Beck’s Depression Inventory (BDI), the Hamilton Assessment of Depression Scale (HAM-D), the State Anxiety Inventory (STAI-S) and the Trait Anxiety Inventory (STAI-T). Mean scores and standard deviations (SD) are given for each group. Baseline differences between groups are analysed, with p-values given to indicate statistical significance. For HAM-D, a significant baseline difference was observed (*p* = .002). Post-test results show between-group variation, reflecting the effect of interventions on these scores. Abbreviations: Active tDCS treatment (A), sham tDCS treatment with ICCT (T), active tDCS treatment with ICCT (AT)

### Beck’s depression inventory

#### Mixed-model ANOVA

**Fig. 4:**
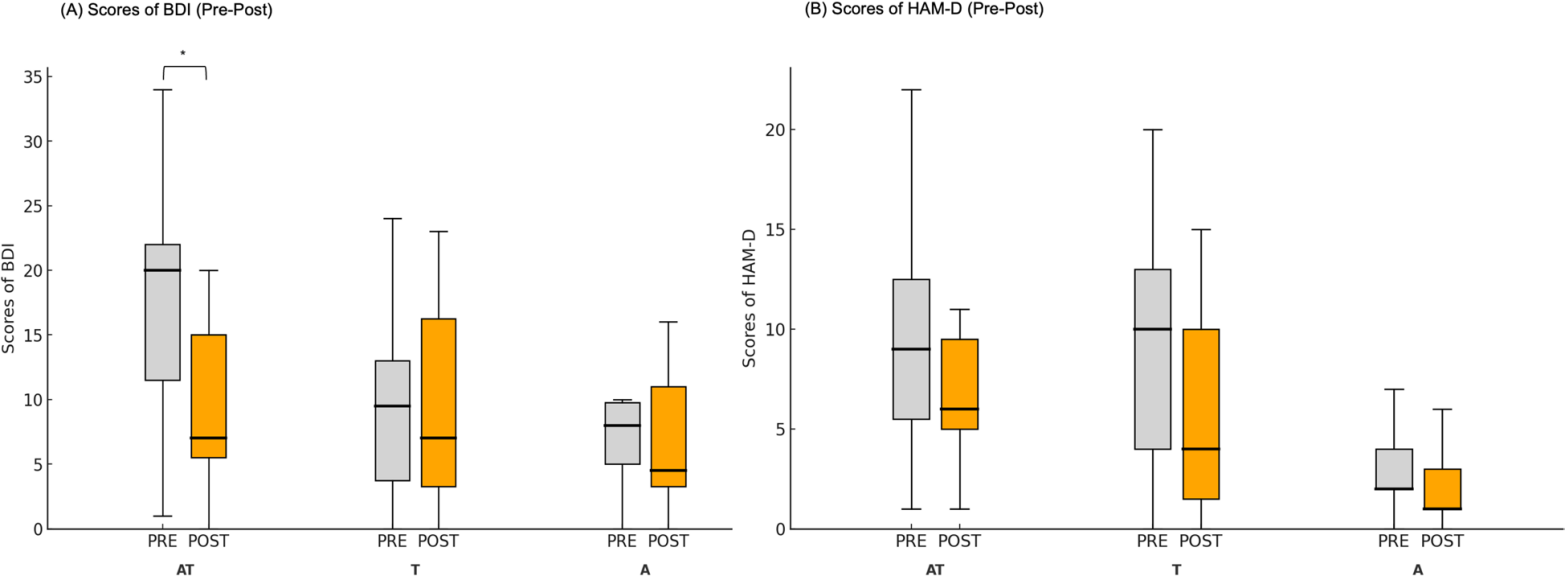
Pre-Post Results of PSD-related scores. The participants were randomly allocated into three groups: A - Active tDCS solely; T - Sham tDCS with ICCT; and AT - Active tDCS with ICCT. **A** For the Beck’s Depression Inventory (BDI), the difference between pre- (gray) and post-testing (orange) was significant in the AT group. **B** For the Hamilton Depression Rating Scale (HAM-D), the treatment effect was globally significant, but no significant difference was found for treatment and conditions. However, we found significant difference at the baseline between A and AT, T groups. (*p < .05)

For depression as measured using the BDI), we found a significant main effect of PRE-POST, F(1,32) = 13.80, *p* < .001, η_p_^2^ = 0.30 and PRE-POST × CONDITION, F(2,32) = 10.80, *p* < .001, η_p_^2^ = 0.40. CONDITION produced no statistically significant contrast between experimental groups, F(2,32) = 1.31, *p* = .285. The Levene’s test showed no significant difference in homogeneity when testing pre- and post-treatment, *p*_pre_ = 0.641; *p*_post_ = 0.751. The post-hoc testing showed a significant difference between AT_pre−post_ conditions, *p* < .001. Results indicate significant improvements in depression over time, with a large effect size, and a treatment effect that varied across groups, particularly favoring the AT group (Fig. 4).

#### Bayesian mixed-model ANOVA

According to the results of Bayesian Mixed-model ANOVA, the “Pre-Post Factor + Condition + Pre-Post Factor * Condition” model has the highest posterior probability and BF10 value, indicating it is the best model for explaining the data. This suggests that the interaction between Pre-Post Factor and Condition is significant. The elevated BFIncl values for each factor and their interaction highlight their significance within the model. The interaction model best explains the variations in BDI scores. Post-hoc comparisons indicate a significant difference between the levels of the pre-post condition, whereas the differences between groups are less prominent. However, the Bayesian ANOVA provided moderate evidence for differences in baseline BDI scores between groups (BF10 = 2.31). The model identified a significant interaction between the Pre-Post Factor and Condition in explaining variations in BDI scores. Additionally, it indicated moderate baseline differences, with the AT group exhibiting higher initial scores (Table 3).

**Table 3:** Results of bayesian repeated measures ANOVA for PSD

Beck’s Depression Inventory (BDI)										
Model Comparison					Post Hoc Comparison					
Models	*P*(M|data)	*P*(Incl|data)	BF10	BFIncl	Levels		Prior Odds	Posterior Odds	BF_10,U_	Error %
Null model	0.00		1.00		**Pre-Post Factor**					
Pre-Post Factor	0.02	0.99	4.28	101.47	Level 1	Level 2	1.00	4.18	4.18	0.00
Condition	0.00	0.98	0.63	30.82	**Condition**					
Pre-Post Factor + Condition	0.01	0.97	2.60	113.17	AT	T	0.59	0.55	0.93	0.01
Pre-Post Factor + Condition + Pre-Post Factor * Condition	0.97		240.69			A	0.59	0.71	1.20	0.01
					T	A	0.59	0.19	0.32	0.01
**Hamilton Depression Rating Scale (HAM-D)**										
Null model	0.01		1.00		**Pre-Post Factor**					
Pre-Post Factor	0.10	0.97	16.44	20.03	Level 1	Level 2	1.00	18.35	18.35	0.00
Condition	0.03	0.90	4.42	5.76	**Condition**					
Pre-Post Factor + Condition	0.69	0.18	115.86	0.89	AT	T	0.59	0.23	0.39	0.01
Pre-Post Factor + Condition + Pre-Post Factor * Condition	0.18		30.50			A	0.59	171.61	292.16	0.00
					T	A	0.59	7.65	13.02	0.00

The Hamilton Depression Rating Scale (HAM-D) and Beck’s Depression Inventory (BDI) outcomes of the Bayesian Repeated Measures ANOVA are summarized in the table. The models assess the effects of Pre-Post Factor, Condition, and their interaction on scores, reporting posterior probabilities P(M∣data), inclusion probabilities P(Incl∣data), Bayes factors (BF10), and inclusion Bayes factors (BFIncl). For BDI, the most robust model includes the Pre-Post Factor and Condition interaction (BF10 = 240.69), while the Pre-Post Factor model for HAM-D shows significant effects (BF10 = 115.86). Post-hoc comparisons reveal pairwise group differences using adjusted posterior odds and Bayes factors. These findings highlight the influence of treatment conditions and pre-post changes on depressive symptoms. Abbreviations: Active tDCS treatment (A), sham tDCS treatment with ICCT (T), active tDCS treatment with ICCT (AT)

### Hamilton depression rating scale

#### Mixed-model ANOVA

On a different measure of depressive symptoms, the HAM-D scale, the main effect of PRE-POST, F(1,32) = 9.46, *p* = .004, η_p_^2^ = 0.23; and the main effect of CONDITION, F(2,32) = 5.10, *p* = .012, η_p_^2^ = 0.25 showed significant differences. However, the PRE-POST × CONDITION interaction did not produce a statistically significant contrast F(2,32) = 0.985, *p* = .385, η_p_^2^ = 0.06. Levene’s test showed a significant difference in homogeneity when testing pre- and post-treatment, p_pre_ = 0.022; p_post_ = 0.020. Results indicate significant improvements in depressive symptoms over time and across groups, with moderate effect sizes, but no significant interaction between time and treatment condition (Fig. 4).

#### Bayesian mixed-model ANOVA

The results of the Bayesian Mixed-model ANOVA show that the “Pre-Post Factor + Condition” model has the highest posterior probability and BF10 value, indicating it is the best model for explaining the data. This suggests that both the Pre-Post and Condition factors significantly impact HAM-D scores. However, the value of BFIncl suggests that while these factors are significant, their interaction is not as strongly supported as their individual effects. The “Pre-Post Factor + Condition + Pre-Post Factor * Condition” model also demonstrates moderate support, however, it is less favored compared to the “Pre-Post Factor + Condition” model. Post-hoc comparisons reveal a significant difference between the levels of the pre-post condition, while the differences between groups are mixed: there is a significant difference between the AT and A groups, but the difference between the T and A groups is less pronounced. The Bayesian ANOVA provided moderate evidence for differences in baseline HAM-D scores between groups, BF10 = 2.85. The Bayesian Mixed-model ANOVA indicates that the Pre-Post and Condition factors significantly impact HAM-D scores, with their interaction showing weaker support. Simultaneously, comparisons reveal significant baseline differences (Table 3).

### Spielberger’s state anxiety inventory

#### Mixed-model ANOVA

**Fig. 5:**
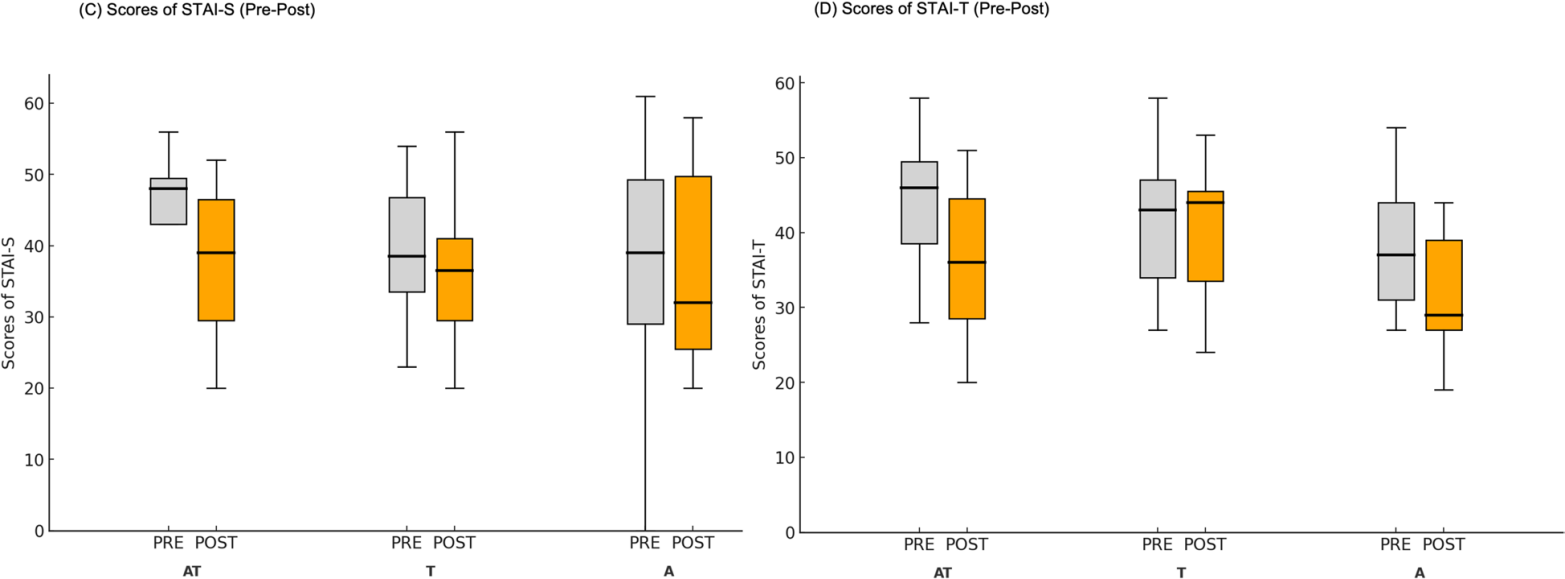
Pre-Post Results of PSA-related scores. The participants were randomly allocated into three groups: A - Active tDCS solely; T - Sham tDCS with ICCT; and AT - Active tDCS with ICCT. (**C**) For Spielberger’s State-Trait Anxiety Inventory - State (STAI-S), the difference between pre- (gray) and post-testing (orange) was not significant, while treatment and conditions showed no significant differences either. **D** The treatment effect was globally significant for Spielberger’s State-Trait Anxiety Inventory - Trait (STAI-T), but no significant difference was found for treatment and conditions. (*p < .05)

One of the anxiety-related scales, the STAI-S, showed a statistically non-significant improvement upon examination. PRE-POST, F(1,32) = 3.706, *p* = .063, η_p_^2^ = 0.10; CONDITION, F(2,32) = 0.482, *p* = .622, η_p_^2^ = 0.03; and PRE-POST × CONDITION interaction, F(2,31) = 0.943, *p* = .400, η_p_^2^ = 0.06. Levene’s test showed no significant difference in homogeneity when testing pre- and post-treatment, p_pre_ = 0.129; p_post_ = 0.099. Thus, our results indicate no statistically significant improvements in anxiety symptoms over time, across groups, or in their interaction, with small effect sizes (Fig. 5).

#### Bayesian mixed-model ANOVA

According to the Bayesian Mixed-model ANOVA, the results show weak support for all models. The “Pre-Post Factor” model has the highest, but still weak, support. The “Condition model” and the “Pre-Post Factor + Condition” model also demonstrates low support, with the “Pre-Post Factor + Condition + Pre-Post Factor * Condition” model exhibiting the least support overall. No significant effects or interactions were found, indicating minimal impact of treatments or time on state anxiety. The results also hold no strong evidence for baseline differences in STAI-S scores between groups, as supported by the results of Bayesian ANOVA, BF10 = 0.35 (Table 4).

**Table 4:** Results of bayesian repeated measures ANOVA for PSA

Spielberger’s State Anxiety Inventory (STAI-S)										
Model Comparison					Post Hoc Comparison					
Models	*P*(M|data)	*P*(Incl|data)	BF10	BFIncl	Levels		Prior Odds	Posterior Odds	BF_10,U_	Error %
Null model	0.31		1.00		**Pre-Post Factor**					
Pre-Post Factor	0.39	0.58	1.26	0.93	Level 1	Level 2	1.00	1.11	1.11	0.02
Condition	0.11	0.31	0.36	0.30	**Condition**					
Pre-Post Factor + Condition	0.15	0.05	0.48	0.22	AT	T	0.59	0.34	0.58	0.01
Pre-Post Factor + Condition + Pre-Post Factor * Condition	0.05		0.17		A		0.59	0.26	0.44	0.01
					T	A	0.59	0.17	0.29	0.01
**Spielberger’s State Anxiety Inventory (STAI-T)**										
Null model	0.16		1.00		**Pre-Post Factor**					
Pre-Post Factor	0.37	0.75	2.33	2.03	Level 1	Level 2	1.00	2.13	2.13	0.00
Condition	0.09	0.47	0.55	0.58	**Condition**					
Pre-Post Factor + Condition	0.21	0.17	1.28	0.84	AT	T	0.59	0.26	0.45	0.01
Pre-Post Factor + Condition + Pre-Post Factor * Condition	0.17		1.09			A	0.59	0.64	1.09	0.01
					T	A	0.59	0.30	0.51	0.01

The Spielberger’s State and Trait Anxiety Inventory (STAI-S and STAI-T) outcomes from Bayesian Repeated Measures ANOVA are summarized in the table. For STAI-S, the Pre-Post Factor model showed moderate support (BF10 = 1.26), while for STAI-T, more robust support was observed (BF10 = 2.33), indicating pre-post changes were notable for trait anxiety. No significant interaction effects or group-level differences were detected, suggesting that changes were primarily associated with time rather than treatment conditions. Abbreviations: Active tDCS treatment (A), sham tDCS treatment with ICCT (T), active tDCS treatment with ICCT (AT).

### Spielberger’s trait anxiety inventory

#### Mixed-model ANOVA

For STAI-T, the main effect of PRE-POST showed a significant decrease, F(1,31) = 6.60, *p* = .015, η_p_^2^ = 0.18. In contrast, the main effect of CONDITION, F(2,31) = 1.23, *p* = .308, η_p_^2^ = 0.07; and PRE-POST × CONDITION interaction, F(2,31) = 2.26, *p* = .121, η_p_^2^ = 0.13, showed no significant effect. Levene’s test showed no significant difference in homogeneity when testing pre- and post-treatment, *p*_pre_ = 0.092; *p*_post_ = 0.813. The results indicate a significant overall decrease in trait anxiety over time, with a moderate effect size, but no significant differences across groups or in the interaction between time and treatment condition (Fig. 5).

#### Bayesian mixed-model ANOVA

The results of Bayesian Mixed-model ANOVA indicate varying levels of support for the models. The “Null model” has low, while the “Pre-Post Factor model” has substantial support, and strong evidence for inclusion. The “Condition model” has low support, indicating moderate evidence for inclusion. The “Pre-Post Factor + Condition” model has the highest support, but weak evidence for inclusion. The “Pre-Post Factor + Condition + Pre-Post Factor * Condition model” shows moderate support. The “Pre-Post Factor + Condition” model has the strongest support, though its inclusion evidence is weaker. Post-hoc comparisons indicate no significant differences between the levels of Pre-Post Factor or between the conditions, although there is some minor support for a difference between the AT and A groups. Furthermore, the analysis indicates no strong evidence for baseline differences in STAI-T scores between groups, BF10 = 0.62. The analysis indicates no strong evidence for baseline differences in STAI-T scores between groups, BF10 = 0.62. The significant PRE-POST effect improves trait anxiety over time, but the non-significant interaction suggests this was not treatment-specific. Minor post-hoc support was found for improvements in the AT group (Table 4).

#### Minimum clinically important differences

The findings from both MCID criteria highlight significant differences among the groups, demonstrating the effectiveness of the combined intervention of active tDCS and ICCT. The first MCID criterion, which accounted for absolute and relative changes, revealed that the AT group had a significantly higher proportion of participants achieving clinically meaningful improvements, χ2 = 10.14, *p* = .006. The second MCID criterion, focusing solely on relative changes, also showed a statistically significant advantage for the AT group, χ2 = 8.43, *p* = .015. This further emphasizes that the combined intervention consistently outperformed the individual interventions (A and T groups) (Supplementary Table 2 A and B).

## Discussion

In this study, we investigated the effects of tDCS and CCT on PSD and PSA in post-stroke patients. For tDCS, we applied anodal stimulation targeting the bilateral frontal areas and DLPFC, extending to the parietal regions. The CCT program was specifically designed to enhance executive functions, focusing on training inhibitory control. Our findings revealed significant improvements in scores for the BDI in the AT group;. However, no significant interactions were observed for the HAM-D, STAI-T, or STAI-S. While our initial hypothesis—that active tDCS would improve mood symptoms as reflected in the questionnaire scores—was partially supported, no significant effect was observed in the group receiving only active tDCS treatment. This is in line with the results of a recent systematic review and meta-analysis, which highlighted that although tDCS can lead to significant improvements in depressive and anxiety symptoms, the effects are often more pronounced when combined with other interventions [46]. Furthermore, a randomised controlled trial demonstrated the safety and efficacy of tDCS in the treatment of post-stroke depression, particularly with left DLPFC stimulation, overall suggesting that specific protocols may yield better results in this area [47]. Our further hypothesis that CCT could increase the effectiveness of tDCS therapy was also only partially confirmed. We found a decrease in PSD-related test scores similar to Li and colleague’s (2022) findings. Concerning PSA, we see similar trends to previous research, such as the meta-analysis of Li and colleagues (2022) and Kulshresthaa and colleagues (2022), which suggest that the changes are less prominent. Our secondary hypothesis, that CCT could enhance the effectiveness of tDCS therapy, was also only partially supported. We found a decrease in PSD-related test scores similar to Li and colleagues (2022) previous findings. Regarding PSA, we observed similar trends to those reported in previous research, including the meta-analyses by Li et al. (2022) and Kulshrestha et al. (2022), which indicate that the changes are less pronounced. It is essential to highlight that a more pronounced benefit was seen when subjects received cognitive training combined with tDCS. Although we detected a statistically significant improvement in BDI test scores in the AT group, the clinical relevance of the results should be considered with caution. While there were no significant baseline differences in BDI test scores, the significant decrease in the initial score of the AT group can be explained by the fact that members of the AT group tended to present with a higher initial BDI score compared to the A and T groups. Thus, a decrease may appear more pronounced, and this does not necessarily imply a clear advantage for the AT group, therefore the results should be treated with critical caution. The observed advantage in the AT group may be influenced by baseline differences and other factors not accounted for in this analysis, again reinforcing the need for careful consideration of these results within the broader study context. Nevertheless, it is possible that the combination of CCT and tDCS might have an additive effect that results in more easily detectable changes. This assumption is further supported by the work of Martin and colleagues (2014), which emphasizes that the “online” training approach we applied (where tDCS and ICCT were administered simultaneously) may be more advantageous compared to “offline” training, where these procedures are applied sequentially. Our analysis further confirms the observed trends, indicating that participants in the AT group were significantly more likely to achieve clinically significant improvement according to the criteria for minimal clinically important difference (MCID) than those in groups A and T. The MCID analysis showed that 70% of participants in the AT group met the combined absolute and relative change criteria (MCID I) and 82% met the relative-only criteria (MCID II), which not only exceeds the statistically significant improvement, but also the clinically significant thresholds. These results suggest that the combination of active tDCS and ICCT provides significant benefits in achieving therapeutic outcomes. Importantly, the high proportion of participants meeting the MCID criteria strongly supports the use of this combined approach in everyday clinical practice to treat depressive symptoms in post-stroke patients. By demonstrating greater efficacy than either treatment alone, this intervention highlights the synergistic potential of integrating cognitive and neuromodulatory therapies into routine neurorehabilitation protocols, offering a practical and effective solution to improve patient care. These considerations also highlight the importance of using MCID thresholds to complement statistical analyses.

We further hypothesized that symptoms of depression and anxiety are not solely linked to dysfunction in the DLPFC but are also associated with a broader cortical network encompassing the prefrontal and parietal regions of the brain. We employed a setup designed to encompass these three key areas as effectively as possible. This was presumably achieved through the selection of the stimulation site and consideration of modeling. However, the results did not yield any direct evidence to support the hypothesis. The decision to target the anodal stimulation point at the AFz area was intentional, guided by prior research and the anticipated benefits of stimulating the frontoparietal network (FPN). However, while Razza and colleagues (2020) found that anodal stimulation of the F3 area was generally effective for treating depression, research by Kaiser and colleagues (2015) and Sylvester and colleagues (2012) suggests that mood-related symptoms may not be solely tied to the DLPFC but rather to a broader dysfunction within the FPN. This hypothesis is supported by Kaiser et al.‘s (2015) meta-analysis, which identified reduced connectivity within the FPN in individuals with depression, based on data from 25 publications. This network dysfunction underscores the potential benefits of targeting the FPN for therapeutic interventions [48]. Consequently, we chose to target the AFz area to engage the broader FPN network. This approach contrasts with the F3 area stimulation, which focuses predominantly on the frontal regions. However, the results did not confirm the effectiveness of this alternative stimulation target. This suggests that while the DLPFC is not the only region involved in mood modulation, and particularly in the development of depressive complaints, neuromodulation may be more effective when targeting the DLPFC rather than the broader FPN. Adding to the complexity is a meta-analysis of Li and colleagues (2022), reporting post-stroke patients to generally benefit from tDCS treatment, regardless of the stimulation target, i.e. DLPFC vs. primary motor cortex.

The methodological variations across studies employing tDCS may account for the inconsistent outcomes observed in tDCS therapy. These differences include variations in sample characteristics and treatment frequency, session duration, current intensity, electrode size, and electrode placement[49]. Studies in the field set the duration of stimulation between 5 and 30 min, with the intensity of the current typically ranging between 1 and 3 mA. For cognitive rehabilitation purposes, the most widely recommended safe current intensity is 2 mA, and electrodes of sizes between 25 cm² and 35 cm² are considered most appropriate [50–52]. In the current research, we strictly adhered to these guidelines by delivering 2 mA of stimulation for 20 min in 10 sessions. This kind of protocol is in line with cognitive rehabilitation parameters. Thus, our results are similar to those of the previous studies in this area. In the current study, we strictly adhered to established guidelines, delivering 2 mA of stimulation for 20 min across 10 sessions. This protocol aligns with standard cognitive rehabilitation parameters. Consequently, our findings are consistent with those reported in previous studies in this field. However, the exact placement of the cathode electrodes was intentionally adjusted to ensure a broader stimulation area. Although this setting was intended to optimize the electric field distribution, it may have limited the desired effect on mood-related symptoms. This consideration raises important questions about the relationship between electrode placement and the underlying neural mechanisms targeted. The connection between depression and anxiety, frequently emphasized in transdiagnostic approaches, suggest that interventions targeting cognitive control (CC) could have an impact on both conditions. Structural network dysfunctions, particularly within the fronto-parietal network (FPN), are known to impair CC, which is essential for regulating thoughts and emotions in a context-appropriate manner. Overall, our results may be in line with the work of Menon and D’Esposito (2022), which identified six major brain networks involved in CC, namely the Salience Network, Cingulo-opercular Network, Ventral Attention Network, Dorsal Attention Network, Default-mode Network, and the FPN. Their research highlights that, while the FPN is a crucial subnetwork, it is only one of several key networks involved. This suggests that targeting the FPN exclusively may not produce substantial effects. In summary, although the guidelines for stimulation intensity and duration were adhered to, it is possible that certain networks critical to the desired outcomes remained outside the range of stimulation.

While the use of tDCS is more extensively researched for treating PSD, the mechanism of action for the CCT in PSD requires further research and understanding. Nevertheless, CCT remains a promising, accessible and practical therapeutic approach for the treatment of PSD. By enhancing patients’ cognitive function, CCT may indirectly alleviate PSD symptoms, contributing to overall improvement in patient condition [53, 54]. Nie and colleagues’ (2022) analysis found that CCT significantly enhanced cognitive functioning following stroke; and while not directly influencing affective components, it may improve perceived quality of life through the enhancement of cognitive skills, ultimately also leading to mood improvements [55]. CCT methods are available in various forms, such as memory, attention, and language therapy, and may influence multiple pathways. It should also be noted that understanding CCT related pathways might be more challenging in post-stroke conditions, due to the prominent impairment in the regulation of cognitive control post-stroke; and CCT interventions are likely more effective in individuals with no neurological condition Further research is warranted to determine the optimal type of CCT and especially for modulating affective components. In summary, the use of CCTs for affective modulation remains underexplored in neurological disorders like stroke. In contrast, results from studies involving non-neurological disorders show variable outcomes. However, our research supports the hypothesis that modulation of anxiety and depressive symptoms through CCT for cognitive control does not lead to significant changes in post-stroke patients.

Regarding anxiety components, recent research aligns with our findings showing no support for the efficacy of tDCS in modulating anxiety symptoms. However, in those studies, the DLPFC was the primary site of stimulation in healthy individuals. Similar conclusions are drawn in a review by Stein et al. (2020), which only interprets the therapeutic efficacy of tDCS when combined with pharmacotherapy and also highlights the lack of research on tDCS modulation of anxiety symptoms [52]. In summary, for depression-related symptoms, more intense stimulation of the brain region primarily responsible for CC, i.e. theDLPFC may be more relevant for tDCS than stimulating a broader brain network with a comparable effect. However, for anxiety complaints, neither the research data to date nor our study supports the efficacy of tDCS when used alone, without additional intervention. It is important to note that research on tDCS for post-stroke anxiety is limited, as it is a relatively recent area of investigation.

Furthermore, while there is less research on PSA compared to PSD, existing studies suggest that tDCS may be more effective in treating anxiety symptoms independently of stroke, particularly when combined with pharmacotherapy and cognitive behavioral therapy [56]. Our findings are consistent with the broader literature on the relationship between PSA and PSD as highlighted in a recent systematic review by Wright and colleagues (2017) [57]. Their work underlines the prevalence of PSA, which affects approximately 24% of stroke survivors, and is strongly associated with PSD, which has an odds ratio of 4.66. This further supports the possibility of a transdiagnostic approach, suggesting that interventions targeting depression may also be effective in alleviating anxiety symptoms in post-stroke populations. However, our study found that while tDCS and CCT were effective in improving depressive symptoms, these interventions did not lead to significant changes in anxiety-related measures (STAI-S, STAI-T). One potential explanation is that anxiety symptoms are associated with more diffuse and variable neural mechanisms compared to PSD, as Wang and colleagues (2021) have highlighted [58]. They found that the relationship between PSA and specific brain regions is not clearly identifiable. This result is consistent with the findings of Chun et al. (2018) and Burton et al. (2013), who also reported no clear correlation between brain region activity and PSA [59, 60]. This may also explain why research has predominantly focused on PSD, as the brain networks involved are more clearly defined based on current understanding. However, Kaiser et al. (2015) and Sylvester et al. (2012), emphasized the role of FPN in mood and anxiety disorders, but it has to be noted that their studies were not specific to post-stroke populations. One could also hypothesize that PSA may indeed be less dependent on frontal processing, which could explain why its treatment with traditional psychotherapeutic methods tends to be less effective [57]. Nevertheless, the correlational methods we examined revealed a significant comorbidity between PSA and PSD symptoms. Therefore, our study confirmed the hypothesis that these two conditions frequently co-occur and may be interconnected. Methodological variability across studies on PSA, including differences in patient populations, assessment tools, and treatment strategies, may partially explain the inconclusive findings. These challenges underscore the need for targeted research into PSA-specific interventions, which may include integrating tDCS with psychotherapeutic or pharmacological approaches.

### Limitations

The limitations of our research may be related to the interpretability of the results. First, the sample we studied could be more interpretable if it were more specifically defined for a post-stroke population, as we worked with a relatively heterogeneous sample in this regard. Certainly, PSD and PSA symptoms may manifest differently in less heterogeneous samples, depending on factors such as the location of the lesion or the time elapsed since the stroke. In addition, the relatively small and unbalanced sample size may limit the generalizability of our findings by reducing statistical power and the ability to detect small effects. Increasing the sample size may yield different results, potentially offering more robust insights. Furthermore, anxiety symptoms did not reach a clinically relevant threshold; consequently, the therapeutic effectiveness of rehabilitation tools in this domain could not be demonstrated. Another limitation is the lack of long-term follow-up, which prevents us from assessing whether the observed effects are sustained over time. Future research should focus on studies with larger and more diverse samples to explore post-stroke mood symptoms. Additionally, incorporating longer follow-up periods would help confirm the durability of the observed effects and provide a more comprehensive understanding of the therapeutic potential of tDCS and ICCT.

## Conclusion

Our results suggest that combining tDCS stimulation with ICCT may improve PSD symptoms after a stroke. However, the clinical interpretation of these results require further critical consideration. Additionally, our findings indicate that this approach does not appear to affectPSA symptoms. To provide more targeted and effective treatment for post-stroke patients in the future, additional studies are needed, particularly focusing on the areas of tDCS stimulation and the type of CCT used.

## Supplementary Information

Additional file 1.

Additional file 2.

## Data Availability

No datasets were generated or analysed during the current study.

## Abbreviations

tDCS: transcranial direct current stimulation
CCT: computer-based cognitive training
ICCT: inhibitory control training
BDI: Beck’s Depression Inventory
HAM-D: Hamilton Depression Rating Scale
State: Spielberger’s State-Trait Anxiety Inventory STAI-S
Trait: Spielberger’s State-Trait Anxiety Inventory
STAI-T: Post-stroke Depression
FPN: Fronto-parietal Network
CC: Cognitive control
