# Supplementary material for: The effect of transcranial direct current stimulation and inhibitory control training on depression and anxiety among post-stroke individuals
Source: BMC Neurol. 2025 Jan 27;25:38. doi: 10.1186/s12883-025-04042-6 (PMC11770995; doi:10.1186/s12883-025-04042-6)
Supplement: Supplementary file 1 — Additional file 1. [file 12883_2025_4042_MOESM1_ESM.docx]

| **Supplementary Table 1.: Correlation Matrix for Pre- and Post-Treatment Measures Across Depression and Anxiety Scales** | | | | | | | | | | | | | | | | | | |  |
| --- | --- | --- | --- | --- | --- | --- | --- | --- | --- | --- | --- | --- | --- | --- | --- | --- | --- | --- | --- |
|  | |  | | **Pre BDI** | | **Pre STAI-S** | | **Pre STAI-T** | | **Pre HAM-D** | | **Post BDI** | | **Post STAI-S** | | **Post STAI-T** | | **Post HAM-D** |  |
| **Pre BDI** |  | r |  |  |  |  |  |  |  |  |  |  |  |  |  |  |  |  |  |
|  |  | df |  |  |  |  |  |  |  |  |  |  |  |  |  |  |  |  |  |
|  |  | p-value |  |  |  |  |  |  |  |  |  |  |  |  |  |  |  |  |  |
| **Pre STAI-S** |  | r |  | 0.60 |  |  |  |  |  |  |  |  |  |  |  |  |  |  |  |
|  |  | df |  | 33 |  |  |  |  |  |  |  |  |  |  |  |  |  |  |  |
|  |  | p-value |  | < .001 |  |  |  |  |  |  |  |  |  |  |  |  |  |  |  |
| **Pre STAI-T** |  | r |  | 0.57 |  | 0.86 |  |  |  |  |  |  |  |  |  |  |  |  |  |
|  |  | df |  | 32 |  | 32 |  |  |  |  |  |  |  |  |  |  |  |  |  |
|  |  | p-value |  | < .001 |  | < .001 |  |  |  |  |  |  |  |  |  |  |  |  |  |
| **Pre HAM-D** |  | r |  | 0.58 |  | 0.50 |  | 0.35 |  |  |  |  |  |  |  |  |  |  |  |
|  |  | df |  | 32 |  | 32 |  | 31 |  |  |  |  |  |  |  |  |  |  |  |
|  |  | p-value |  | < .001 |  | 0.003 |  | 0.043 |  |  |  |  |  |  |  |  |  |  |  |
| **Post BDI** |  | r |  | 0.75 |  | 0.55 |  | 0.43 |  | 0.58 |  |  |  |  |  |  |  |  |  |
|  |  | df |  | 33 |  | 33 |  | 32 |  | 32 |  |  |  |  |  |  |  |  |  |
|  |  | p-value |  | < .001 |  | < .001 |  | 0.010 |  | < .001 |  |  |  |  |  |  |  |  |  |
| **Post STAI-S** |  | r |  | 0.45 |  | 0.61 |  | 0.54 |  | 0.39 |  | 0.56 |  |  |  |  |  |  |  |
|  |  | df |  | 33 |  | 33 |  | 32 |  | 32 |  | 33 |  |  |  |  |  |  |  |
|  |  | p-value |  | 0.007 |  | < .001 |  | 0.001 |  | 0.021 |  | < .001 |  |  |  |  |  |  |  |
| **Post STAI-T** |  | r |  | 0.68 |  | 0.75 |  | 0.65 |  | 0.71 |  | 0.79 |  | 0.64 |  |  |  |  |  |
|  |  | df |  | 33 |  | 33 |  | 32 |  | 32 |  | 33 |  | 33 |  |  |  |  |  |
|  |  | p-value |  | < .001 |  | < .001 |  | < .001 |  | < .001 |  | < .001 |  | < .001 |  |  |  |  |  |
| **Post HAM-D** |  | r |  | 0.54 |  | 0.47 |  | 0.28 |  | 0.68 |  | 0.60 |  | 0.35 |  | 0.58 |  |  |  |
|  |  | df |  | 33 |  | 33 |  | 32 |  | 32 |  | 33 |  | 33 |  | 33 |  |  |  |
|  |  | p-value |  | < .001 |  | 0.004 |  | 0.106 |  | < .001 |  | < .001 |  | 0.040 |  | < .001 |  |  |  |

The table presents the Pearson correlation coefficients and p-values for relationships between pre-and post-treatment measures of depression and anxiety scales, including the Beck Depression Inventory (BDI), State-Trait Anxiety Inventory (STAI-S and STAI-T), and Hamilton Depression Rating Scale (HAM-D). Strong correlations were observed across several measures, particularly between Post STAI-T and Post BDI (r=.79,p<.001) and between Pre BDI and Post BDI (r=.75,p<.001). These findings highlight consistent relationships between depressive and anxiety-related outcomes before and after treatment.
