# Supplementary material for: The effect of transcranial direct current stimulation and inhibitory control training on depression and anxiety among post-stroke individuals
Source: BMC Neurol. 2025 Jan 27;25:38. doi: 10.1186/s12883-025-04042-6 (PMC11770995; doi:10.1186/s12883-025-04042-6)
Supplement: Supplementary file 2 — Additional file 2. [file 12883_2025_4042_MOESM2_ESM.docx]

| **Supplementary Table 1. A.: Proportion of Participants Meeting MCID Criteria Across Groups for BDI** | | | | | | | | |  | |
| --- | --- | --- | --- | --- | --- | --- | --- | --- | --- | --- |
| Subject | Group | Pre-Post Change | Absolute value of change | BDI change >5 | BDI change > 29.64% | Direction | MCID Criterion I. | MCID Criterion II. | |  |
| 1,00 | T | -1,00 | 1,00 | no | 0,04 | improved | no | no | |  |
| 2,00 | T | 1,00 | 1,00 | no | 0,50 | deteriorated | no | no | |  |
| 3,00 | T | 0,00 | 0,00 | no | 0,00 | unaltered | no | no | |  |
| 4,00 | T | 4,00 | 4,00 | no | 0,50 | deteriorated | no | no | |  |
| 5,00 | T | -3,00 | 3,00 | no | 0,43 | improved | no | yes | |  |
| 6,00 | T | 10,00 | 10,00 | yes | 0,77 | deteriorated | no | no | |  |
| 7,00 | T | -4,00 | 4,00 | no | 0,36 | improved | no | yes | |  |
| 8,00 | T | -6,00 | 6,00 | yes | 0,46 | improved | yes | yes | |  |
| 9,00 | T | 2,00 | 2,00 | no | 0,13 | deteriorated | no | no | |  |
| 10,00 | T | 0,00 | 0,00 | no | 0,00 | deteriorated | no | no | |  |
| 11,00 | T | 8,00 | 8,00 | yes | 0,73 | deteriorated | no | no | |  |
| 12,00 | T | -2,00 | 2,00 | no | 1,00 | improved | no | yes | |  |
| 13,00 | T | 0,00 | 0,00 | no | 0,00 | unaltered | no | no | |  |
| 14,00 | T | 0,00 | 0,00 | no | 1,00 | unaltered | no | no | |  |
| 15,00 | A | -3,00 | 3,00 | no | 0,11 | improved | no | no | |  |
| 16,00 | A | 5,00 | 5,00 | no | 0,71 | deteriorated | no | no | |  |
| 17,00 | A | -6,00 | 6,00 | yes | 0,60 | improved | yes | yes | |  |
| 18,00 | A | -5,00 | 5,00 | no | 1,00 | improved | no | yes | |  |
| 19,00 | A | -6,00 | 6,00 | yes | 0,67 | improved | yes | yes | |  |
| 20,00 | A | 0,00 | 0,00 | no | 1,00 | unaltered | no | no | |  |
| 21,00 | A | 0,00 | 0,00 | no | 0,00 | unaltered | no | no | |  |
| 22,00 | A | 4,00 | 4,00 | no | 1,00 | deteriorated | no | no | |  |
| 23,00 | A | -1,00 | 1,00 | no | 0,11 | improved | no | no | |  |
| 24,00 | A | -4,00 | 4,00 | no | 0,20 | improved | no | no | |  |
| 25,00 | AT | -5,00 | 5,00 | no | 0,24 | improved | no | no | |  |
| 26,00 | AT | -1,00 | 1,00 | no | 1,00 | improved | no | yes | |  |
| 27,00 | AT | -11,00 | 11,00 | yes | 0,46 | improved | yes | yes | |  |
| 28,00 | AT | -8,00 | 8,00 | yes | 0,53 | improved | yes | yes | |  |
| 29,00 | AT | -8,00 | 8,00 | yes | 0,36 | improved | yes | yes | |  |
| 30,00 | AT | -9,00 | 9,00 | yes | 1,00 | improved | yes | yes | |  |
| 31,00 | AT | 0,00 | 0,00 | no | 0,00 | unaltered | no | no | |  |
| 32,00 | AT | -17,00 | 17,00 | yes | 0,50 | improved | yes | yes | |  |
| 33,00 | AT | -7,00 | 7,00 | yes | 0,54 | improved | yes | yes | |  |
| 34,00 | AT | -4,00 | 4,00 | no | 0,40 | improved | no | yes | |  |
| 35,00 | AT | -17,00 | 17,00 | yes | 0,77 | improved | yes | yes | |  |
| Table of criteria used to define the minimum clinically important difference (MCID). It includes thresholds for the direction of improvement, absolute change in score, and percentage of baseline score, as well as data for each participant used to assess whether these criteria were met. Active tDCS treatment (A), sham tDCS treatment with ICCT (T), active tDCS treatment with ICCT (AT); Beck’s Depression Inventory (BDI). MCID Criterion I.: Assesses meaningful improvement by considering both BDI change >5 and BDI change > 29.64% in participants' scores; MCID Criterion II.: Assesses meaningful improvement by considering only BDI change > 29.64% in participants' scores | | | | | | | | | | |

| **Supplementary Table 1. B.: Results of χ2-tests for MCID I. and MCID II.** | | | | | | | |
| --- | --- | --- | --- | --- | --- | --- | --- |
|  |  | MCID I. Criterion | | | MCID II. Criterion | | |
| Group | | Not exceeded | Exceeded | N | Not Exceeded | Exceeded | N |
| A | Observed | 8 | 2 | 10 | 7 | 3 | 10 |
|  | Expected | 7.14 | 2.86 | 10.00 | 5.43 | 4.57 | 10.00 |
| AT | Observed | 4 | 7 | 11 | 2 | 9 | 11 |
|  | Expected | 7.86 | 3.14 | 11.00 | 5.97 | 5.03 | 11.00 |
| T | Observed | 13 | 1 | 14 | 10 | 4 | 14 |
|  | Expected | 10.00 | 4.00 | 14.00 | 7.60 | 6.40 | 14.00 |
|  |  |  |  |  |  |  |  |
|  | χ2-tests | Value | df | p | Value | df | p |
|  |  | 10.14 | 2 | 0.006 | 8.43 | 2 | 0.015 |
| The table presents the results of χ²-tests for MCID Criteria I and II across the three groups. Both tests reveal statistically significant differences in the proportion of participants meeting the MCID criteria, with the AT group showing the highest number of participants achieving clinically meaningful improvement. Active tDCS treatment (A), sham tDCS treatment with ICCT (T), active tDCS treatment with ICCT (AT); Beck’s Depression Inventory (BDI). MCID Criterion I.: Assesses meaningful improvement by considering both BDI change >5 and BDI change > 29.64% in participants' scores; MCID Criterion II.: Assesses meaningful improvement by considering only BDI change > 29.64% in participants' scores | | | | | | | |
|  |  |  |  |  |  |  |  |
